# Three genes encoding AOP2, a protein involved in aliphatic glucosinolate biosynthesis, are differentially expressed in *Brassica rapa*

**DOI:** 10.1093/jxb/erv331

**Published:** 2015-07-17

**Authors:** Jifang Zhang, Zhiyuan Liu, Jianli Liang, Jian Wu, Feng Cheng, Xiaowu Wang

**Affiliations:** Institute of Vegetables and Flowers, Chinese Academy of Agricultural Sciences, Zhongguancun Nandajie No. 12, Haidian District, Beijing 100081, PR China

**Keywords:** *Brassica rapa*, *BrAOP2*, expression pattern, functional divergence, glucosinolates, paralogues.

## Abstract

The glucosinolate biosynthetic gene *AOP2* encodes an enzyme that plays a crucial role in catalysing the conversion of beneficial glucosinolates into anti-nutritional ones. In *Brassica rapa*, three copies of *BrAOP2* have been identified, but their function in establishing the glucosinolate content of *B. rapa* is poorly understood. Here, we used phylogenetic and gene structure analyses to show that BrAOP2 proteins have evolved via a duplication process retaining two highly conserved domains at the N-terminal and C-terminal regions, while the middle part has experienced structural divergence. Heterologous expression and *in vitro* enzyme assays and *Arabidopsis* mutant complementation studies showed that all three *BrAOP2* genes encode functional BrAOP2 proteins that convert the precursor methylsulfinyl alkyl glucosinolate to the alkenyl form. Site-directed mutagenesis showed that His356, Asp310, and Arg376 residues are required for the catalytic activity of one of the BrAOP2 proteins (BrAOP2.1). Promoter–β-glucuronidase lines revealed that the *BrAOP2.3* gene displayed an overlapping but distinct tissue- and cell-specific expression profile compared with that of the *BrAOP2.1* and *BrAOP2.2* genes. Quantitative real-time reverse transcription-PCR assays demonstrated that *BrAOP2.1* showed a slightly different pattern of expression in below-ground tissue at the seedling stage and in the silique at the reproductive stage compared with *BrAOP2.2* and *BrAOP2.3* genes in *B. rapa*. Taken together, our results revealed that all three *BrAOP2* paralogues are active in *B. rapa* but have functionally diverged.

## Highlight

The characterization of three *BrAOP2* paralogues, crucial candidates for engineering beneficial glucosinolates, revealed that functional divergence occurred during the evolution of triplicated *AOP2* genes in *Brassica rapa*.

## Introduction

Glucosinolates are a class of nitrogen- and sulfur-rich secondary metabolites common in the family Brassicaceae (or Cruciferae), which includes the model plant *Arabidopsis thaliana* and economically important *Brassica* crops such as turnip (*Brassica rapa* ssp. *rapa*), broccoli (*Brassica oleracea* var. italica), and cauliflower (*B*. *oleracea* var. botrytis) (Halkier and Gershenzon, 2006). Glucosinolates and their breakdown products have been the subject of extensive studies because of their roles in plant defence against microbial pathogens and herbivorous insects (Kroymann *et al.*, 2003; Clay *et al.*, 2009).

Some glucosinolates and their breakdown products act as anti-carcinogenic compounds. For example, Met-derived 4-methylsulfinylbutyl glucosinolate [glucoraphanin (GRA)] has been the focus of considerable attention because its degradation product, sulforaphane, which was first isolated from broccoli, exhibits pronounced anti-carcinogenic activity (Zhang *et al.*, 1994; Fahey *et al.*, 1997; Gamet-Payrastre *et al.*, 2000; Talalay and Fahey, 2001; Fahey *et al.*, 2002). However, sulforaphane can undergo further reactions to generate 2-hydroxy-3-butenyl glucosinolate (progoitrin), which is goitrogenic and lacks notable anti-cancer activity (Faulkner *et al.*, 1998). Thus, it will be advantageous to manipulate glucosinolate structures to alter the nutritional and economic value of *Brassica* vegetables.

Glucosinolates are derived from glucose and an amino acid, and can be classified as aliphatic, aromatic, or indole glucosinolates based on the precursor amino acid (Gershenzon, 2006; Sonderby *et al.*, 2010). Their biosynthesis occurs in three independent stages: (i) side-chain elongation of the precursor amino acid; (ii) formation of the core structure; and (iii) modification of the side chain. Elongation and modification of the side chain (R-group) generates diverse glucosinolate compounds, of which more than 200 structures have been identified (Clarke, 2010).

In *Arabidopsis thaliana*, a small gene family of 2-oxoglutarate-dependent dioxygenases (*AtAOP1*, *AtAOP2*, and *AtAOP3*) that resulted from gene duplication was identified by fine-scale mapping (Hall *et al.*, 2001; Kliebenstein *et al.*, 2001*b*). A heterologous expression and *in vitro* enzyme assay showed that AOP2 catalysed the conversion of 3-methylsulfinylpropyl- and 4-methylsulfinylbutyl glucosinolates to the corresponding alkenyl glucosinolates 2-propenyl and 3-butenyl, respectively, while AOP3 displayed only weak catalytic activity in the conversion of 3-methylsulfinylpropyl glucosinolate to 3-hydroxypropyl glucosinolate (Kliebenstein *et al.*, 2001*b*). *AtAOP1* is believed to be the ancestral gene that gave rise to *AtAOP2* and *AtAOP3* through a series of gene duplication events; however, its function is unclear (Kliebenstein *et al.*, 2001*b*). *Arabidopsis thaliana* has been reported to display differential *AOP* expression, whereby particular accessions expressed either *AtAOP2* or *AtAOP3* or neither, but not both. Moreover, in some *Arabidopsis* accessions the absence of both functional enzymes has led to the accumulation of the precursor methylsulfinylalkyl glucosinolate (Kliebenstein *et al.*, 2001*a*). The mechanism by which a particular accession transcribes *AtAOP2* or *AtAOP3* but not both was found to involve the complete inversion of the two structural genes resulting in *AtAOP3* being expressed from the *AtAOP2* promoter (Chan *et al.*, 2010; Kliebenstein *et al.*, 2001*b*). *Arabidopsis* accessions have also been shown to have three different *AtAOP2* alleles with variations in exon 2; for example, *AtAOP2* from the Columbia (Col-0) ecotype contains a 5bp frame-shift deletion that led to the accumulation of methylsulfinylalkyl glucosinolates (Kliebenstein *et al.*, 2001*b*; Neal *et al.*, 2010).

*Brassica* crops are of great economical and nutritional importance to humans. In *B. oleracea*, *BoAOP1* and *BoAOP2* were found to be duplicated, while *BoAOP3* was absent (Gao *et al.*, 2004), and the *BoAOP2* homologue (*BoGSL-ALK*) from collard (*B. oleracea* var. viridis) was reported to catalyse the conversion of methylsulfinylalkyl glucosinolate to the alkenyl form in plants (Li and Quiros, 2003). Broccoli (*B. oleracea* var. italica), on the other hand, contains a non-functional allele of *BoGSL-ALK* because of a 2bp deletion in exon 2, which causes the accumulation of GRA (Li and Quiros, 2003). More recently, another non-functional *BoAOP2* gene contributing to the accumulation of GRA because of the presence of a premature stop codon has been found in *B. oleracea* (Liu *et al.*, 2014).

In *B. rapa*, which underwent an additional whole-genome triplication after its divergence from a common ancestor of *Arabidopsis thaliana*, three orthologues of the *BrAOP* loci were found, each containing the tandem duplicated genes *BrAOP1* and *BrAOP2* but not *BrAOP3* (Wang *et al.*, 2011). These duplicated *BrAOP2* genes may enhance the potential resources for quantitative variation of a particular trait (Li *et al.*, 2008). To the best of our knowledge, however, no studies have investigated whether all three *BrAOP2* genes are functional.

Here, we investigated the consequence of polyploidy on *BrAOP2* structure, phylogeny, gene expression, and function both *in vivo* and *in vitro*. Our results highlight the importance of *BrAOP2* paralogues in controlling the conversion of beneficial glucosinolates to harmful ones and the expression divergence of duplicated *BrAOP2* genes. These findings will help in enriching beneficial aliphatic glucosinolates (e.g. GRA) and in reducing anti-nutritional aliphatic glucosinolates (e.g. progoitrin and gluconapin) in *B. rapa* for the benefit of humans.

## Materials and methods

### Plant material

*B. rapa* accessions yellow sarson L143 and Chiifu-401/42 were germinated and grown in greenhouses at the Chinese Academy of Agricultural Sciences (Beijing, China) during the spring of 2011. Leaves were collected from Chiifu-401/42 for cloning of *BrAOP2* genes. Accession L143 plants were used to collect different organs (root, stem, leaves, inflorescence, and siliques) to analyse *BrAOP2* expression patterns. We collected samples of three biological replicates under normal growth conditions 2 and 10 weeks after sowing. Two to three healthy, undamaged fresh leaves were collected, snap frozen in liquid nitrogen, and kept at –80°C until use.

*Arabidopsis thaliana* ecotype Col-0 was used for functional complementation studies *in vivo*. Col-0 seeds were plated on soil and cold treated at 4 °C for 2 d in the dark. After stratification, the seeds were transferred into a temperature-controlled growth chamber at 22 °C, with a 16/8h photoperiod, light intensity of 120 mmol m^−2^ s^−1^, and 40% relative humidity.

The seeds of plants grown on Petri dishes were briefly surface sterilized with 75% (v/v) ethanol for 8min and then washed three times with sterile water. Seeds were sown on Murashige and Skoog (MS) agar medium (half-strength MS salt, pH 5.8) and cold treated at 4 °C for 2 d in the dark, and then placed in the growth chamber. Transgenic plants were selected by germination on half-strength MS medium containing 60 µg ml^−1^ of kanamycin and 30 µg ml^−1^ of hygromycin antibiotics and were treated subsequently as wild-type plants.

### Sources of genome data

*B. rapa* gene sequences for synteny analyses were obtained from BRAD (v.1.5; http://brassicadb.org) (Cheng *et al.*, 2011). *AOP1* and *AOP2* sequences and genome datasets for *Arabidopsis thaliana* were downloaded from The *Arabidopsis* Information Resource (TAIR9; http://www.arabidopsis.org/index.jsp). *Arabidopsis AOP3* sequences were from GenBank (http://www.ncbi.nlm.nih.gov/). The genomic dataset for *Arabidopsis lyrata* was downloaded from the Joint Genome Initiative database (Gene model 6; http://genome.jgi-psf.org/Araly1/Araly1.home.html) (Hu *et al.*, 2011), gene and genome data for *Thellungiella halophila* were from Yang *et al.* (2013), *Schrenkiella parvula* and *Thellungiella salsuginea* datasets were from Dassanayake *et al.* (2011) and Wu *et al.* (2012), *Leavenworthia alabamica*, *Sisymbrium irio*, and *Aethionema arabicum* genomic data sets were from Haudry *et al.* (2013), *Camelina sativa* and *B. oleracea* genomic datasets were from Kagale *et al.* (2014) and Liu *et al.* (2014), and the *Raphanus sativus* genomic dataset was obtained from Kitashiba *et al.* (2014).

### Phylogenetic analysis and motif identification

*AOP* genes from other sequenced Brassicaceae species such as *Arabidopsis lyrata*, *S. parvula*, *T. salsuginea*, *T. halophila*, *L. alabamica*, *S. irio*, *Aethionema arabicum*, *Camelina sativa*, *B. oleracea*, and *R. sativus* were identified based on BRAD multi-syntenic orthologues with *Arabidopsis thaliana* (http://brassicadb.org/brad/searchSyntenytPCK.php). We aligned full-length sequences of AOP proteins from the sequenced Brassicaceae genomes using CLUSTAL W with default parameters (Larkin *et al.*, 2007). The phylogenetic tree was reconstructed based on the conserved sequences in the N-terminal and C-terminal parts of AOP proteins using the neighbour-joining method with MEGA v.6.0 software (Tamura *et al.*, 2011), and bootstrap values with 1000 replicates were calculated. MEME version 4.9.1 (Bailey *et al.*, 2009) was used to identify the conserved motifs of syntenic AOP proteins in the sequenced Brassicaceae species. The parameters for the analysis were as follows: number of repetitions, 0 or 1; maximum number of motifs, 14; and optimum motif width, 6–100. The MAST program (Bailey and Gribskov, 1998) was used to search for each of the motifs in the AOP sequences.

### Heterologous expression and enzyme assays

Total leaf RNA was isolated using a Total RNA Extraction kit according to the manufacturer’s instructions (Sangon, Shanghai, China). First-strand cDNA was synthesized from approximately 2 µg of total RNA using a TransScript First Strand cDNA Synthesis SuperMix kit (TransGen Biotech, Beijing, China) with oligo(dt) as a primer in a 20 µl reaction. Full-length cDNAs of *BrAOP2.1*, *BrAOP2.2*, and *BrAOP2.3* were amplified using gene-specific primers (Supplementary Table S3, available at *JXB* online) and cloned into pET-32a expression vectors (Novagen, Madison, WI, USA). This procedure placed the cDNAs into a fusion protein with thioredoxin under the control of a T7 promoter. After cloning, the inserted cDNA was sequenced to verify the junctions and to ensure that no mutations were introduced by PCR. The construct was expressed in *Escherichia coli* strain BL21(DE3) grown in Luria–Bertani medium to an OD of 0.6 at 600nm. Induction of recombinant protein synthesis was initiated by the addition of 0.5mM isopropyl β-d-thiogalactopyranoside overnight at 16 °C. Cells were harvested by centrifugation at 8000*g*. After resuspension in 1M Tris/HCl (pH 7.5), 200mg of lysozyme was added and incubated on ice for 30min. The cells were then sonicated twice with a microprobe at 55% of full power for a 5min 20% cycle. Cell debris was precipitated by centrifugation at 12 000*g* and the supernatant was used for enzyme purification by the BugBuster^®^ Ni-NTA His Bind Purification kit following the manufacturer’s instructions (Novagen). The purified lysate was tested by SDS-PAGE and then used for the enzyme assays.

Enzyme assays were conducted as described by Kliebenstein *et al.* (2001*b*) with some modifications. The assay mixture contained 200mM sucrose, 10mM oxoglutarate, 10mM ascorbate, 100 µl of purified intact GRA, 200 µM FeSO_4_, and 400 µl of enzyme preparation in a final volume of 4ml. The reactions were allowed to proceed for 4h at 28 °C, and the glucosinolates were then purified and analysed by high-performance liquid chromatography (HPLC).

### Subcellular localization of BrAOP2 proteins

To identify the subcellular localization of the three BrAOP2 proteins, Pro_CAMV35S_:BrAOP2:GFP (green fluorescent protein) constructs were made. *BrAOP2* coding sequences without stop codons were isolated and cloned into the C-terminal GFP fusion vector pSPYCE-35S/pUC-SPYCE. The resulting constructs were verified by DNA sequencing. The subcellular localization of BrAOP2 proteins was detected by monitoring the transient expression of GFP in *B. rapa* mesophyll protoplast cells (Yoo *et al.*, 2007) under a confocal laser-scanning microscope (Nikon, Tokyo, Japan). GFP fluorescence was imaged at an excitation wavelength of 488nm, and the emission signal was detected at 495–530nm for GFP and at 643–730nm for chlorophyll autofluorescence.

### Site-directed mutagenesis of BrAOP2

To identify the key active-site residues of BrAOP2, site-directed mutagenesis was performed according to the procedure of Ho *et al.* (1989) by overlap extension using PCR. The mutagenic primers used to randomly change two histidines to leucine (H308L and H356L), aspartic acid to alanine (D310A), and arginine to tryptophan (R376W) are shown in Supplementary Table S4, available at *JXB* online. The mutated sequences were analysed by sequencing after being cloned into the pEASY-T1 vector (TransGen Biotech). The heterologous expression and enzyme assays of the mutant proteins were also analysed as described above.

### Generation of transgenic plants overexpressing *BrAOP2*

The coding sequences of *BrAOP2* genes were isolated and amplified using Chiifu-401/42 genomic cDNA as a template with gene-specific primers including restriction sites (*Xba*I/*Sma*I) and ligated to the pEASY-T1 vector. Sequencing analysis was performed and the pEASY-T1:*BrAOP2* constructs were digested with *Xba*I/*Sma*I and inserted into a pBI121 vector driven by the cauliflower mosaic virus (CaMV) 35S promoter. The resulting construct was verified by DNA sequencing and subsequently transformed into *Agrobacterium tumefaciens* (strain GV3101). The binary vector pBI121 contains a kanamycin resistance gene to aid in the selection of transformed *Arabidopsis* lines. The floral infiltration method (Clough and Bent, 1998) was used to transform Col-0 plants. The T1 generation was first screened on kanamycin selection medium (half-strength MS salt, 60mg l^−1^ of kanamycin) and then transferred to soil. The T2 generation derived from selected plants was used to identify homozygous transformed lines. T3 generation homozygous plants were subsequently used in HPLC analysis.

### Histochemical analysis of transgenic plants expressing Pro_BrAOP2_:GUS fusion constructs

The *BrAOP2* promoter regions (~1.2kb) were amplified from the genomic DNA of Chiifu-401/42 plants and cloned into the pEASY-1 vector. To drive β-glucuronidase gene (*GUS*) expression under the control of *BrAOP2* promoters, the plant transformation vectors pBI121 and pCambia 1300 were recombined with *Hin*dIII/*Xba*I (*BrAOP2.1* Pro) and *Pst*I/*Xba*I (*BrAOP2.2* Pro and *BrAOP2.3* Pro) reactions (Supplementary Table S5, available at *JXB* online). The identified Pro_*BrAOP2*_:GUS clones were transformed into *Agrobacterium tumefaciens* strain GV3101 and *Arabidopsis thaliana* Col-0 plants. The histochemical localization of GUS in several independent transgenic lines harbouring the Pro_*BrAOP2*_:GUS construct was performed as described by Jefferson *et al.* (1987) with some modifications. Sample tissues were infiltrated with reaction buffer (50mM Na_2_HPO_4_/NaH_2_PO_4_, pH 7.0, 0.5mM K_3_Fe(CN)_6_, 0.5mM K_4_Fe(CN)_6_, containing 2mM 5-bromo-4-chloro-3-indolyl-β-d-glucuronic acid as substrate), and incubated at 37°C overnight. Plant pigments were destained with 75% ethanol, and GUS staining patterns were recorded under a binocular microscope (Stemi 2000-C; Zeiss).

### Glucosinolate extraction and HPLC analysis

Glucosinolates were extracted and measured as described previously (Hongju and Schnitzler, 2002). Lyophilized samples (0.20g) were weighed accurately in 15ml plastic tubes and immersed in boiling methyl alcohol (5ml) containing 100 µl of benzyl glucosinolate as the internal standard. After 20min of additional shaking, samples were cooled at 4 °C and centrifuged at 3000*g* for 10min. The supernatant fraction (extract) was cleaned twice with 70% methyl alcohol, loaded on DEAE Sephadex A-25 columns and desulfated overnight at room temperature using purified sulfatase (Sigma, E.C. 3.1.6.) prior to HPLC. The column was then washed three times with 0.5ml of deionized water, and the eluent filtrated through a 0.45 µm membrane was used for HPLC analysis. Glucosinolates were identified by comparing retention times and UV absorption spectra with purified standards. The concentration of individual glucosinolates was calculated in nmol mg^−1^ of dry weight relative to the area of the internal standard peak using the respective response factors reported previously (Brown *et al.*, 2003).

### Reverse transcriptase (RT)-mediated first-strand synthesis and real-time quantitative (q)RT-PCR analysis

Total RNA was isolated from different organs using a Total RNA Extraction kit according to the manufacturer’s instructions (Sangon) and then treated with DNase I (Sigma-Aldrich, MO, USA) to eliminate DNA. The RNA purity was determined spectrophotometrically, and the quality was determined by examining rRNA bands on 1% agarose gels. cDNA was synthesized from approximately 2 µg of total RNA using TransScript First-Strand cDNA Synthesis SuperMix (TransGen Biotech) with oligo(dT) as a primer in a 20 µl reaction.

Primer specificity for the three *BrAOP2* genes and *BrGAPDH* was verified by DNA sequencing after cloning the PCR products into the pEASY-T1 vector. The efficiency of gene-specific *BrAOP2* and *BrGAPDH* primers was ascertained initially using a 4-fold serial dilution of L143 cDNA. A linear correlation coefficient (*r* ^2^) of 0.95 and above was observed over a 64-fold dilution range, indicating the high efficiency of each primer pair. qRT-PCR was performed in a total volume of 15 µl, including 2 µl of diluted cDNA, 0.5 µl of each primer (10 pM), and 7.5 µl 2×SYBR Green Master Mixes (Thermo Fisher, MA, USA) on an Eppendorf Real-Time PCR System (Eppendorf, Hamburg, Germany) according to the manufacturer’s instructions. The qRT-PCR program was conducted at 95 °C for 2min, followed by 40 cycles of 95 °C for 30 s and 60°C for 60 s. The expression level of *BrGAPDH* (glyceraldehyde 3-phosphate dehydrogenase) was used as an internal control and the expression of other genes was computed using the 2^–ΔΔ*C*T^ method (Livak and Schmittgen, 2001). Data were analysed from three independent sets of biological replicates with three technical replicates for each. The primers used in this work are listed in Supplementary Table S6, available at *JXB* online.

### Statistical analysis

Data from different experimental sets were analysed for statistical significance using one-way analysis of variance (ANOVA) with a Duncan post-hoc test with SPSS software. A *P* value of <0.05 was considered significant.

## Results

### Paralogous relationship of the three *BrAOP2* genes

**Fig. 1.**
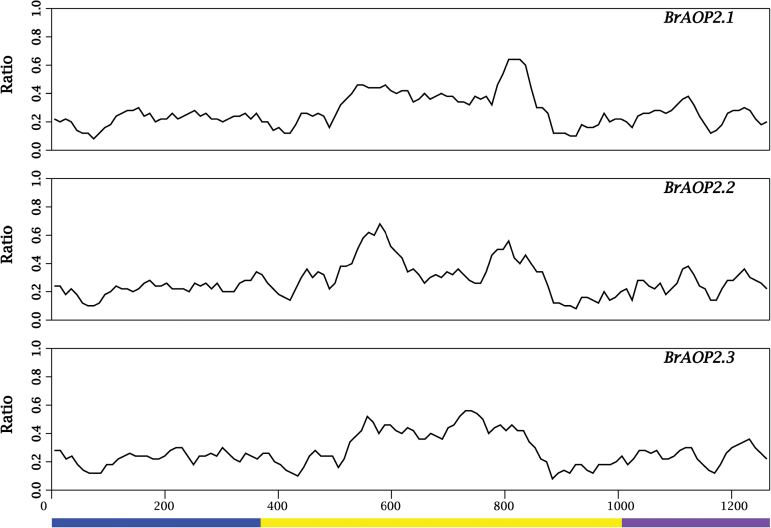
Nucleotide polymorphisms in the three *BrAOP2* and *AtAOP2* sequences. Each *BrAOP2* sequence was compared with the *AtAOP2* sequence, and the relative frequency of nucleotide substitution between them was tested. The vertical axis reflects the relative frequency of nucleotide substitution between *BrAOP2* and *AtAOP2* sequences per sliding window. A sliding window of 50bp with a step width of 10bp was used. Alignment gaps were included in scaling the horizontal axis. The three exons of *AtAOP2* are depicted under the horizontal axis. (This figure is available in colour at *JXB* online.)

In *B. rapa*, three *AOP2* paralogues produced by genome replication and distributed in different chromosomes have been annotated in the Brassica Database (BRAD, http://brassicadb.org); namely, *BrAOP2.1* (Bra03418, A09), *BrAOP2.2* (Bra000848, A03), and *BrAOP2.3* (Bra018521, A02). We isolated and sequenced the *BrAOP2* genes from *B. rapa* accession Chiifu-401/42 based on their sequences in BRAD (Cheng *et al.*, 2011). The three *BrAOP2* genes contain two conserved (exon 1 and exon 3) and one variable (exon 2) exons compared with *AtAOP2* (Fig. 1). As a result, the open reading frames varied from 1287 to 1323bp, encoding proteins of 439, 440, and 428 aa, respectively (Table 1). The deduced amino acid sequences from the three *BrAOP2* genes were 74–81% identical (Supplementary Table S1, available at *JXB* online), and shared high similarity (74–96%) with the functional BoAOP2 protein, and lower similarity (55–60%) with AtAOP2 ecotype Cvi (Supplementary Table S1).

**Table 1.** Summary of the BrAOP2 sequences used in the study

Gene ID	Coding sequence (bp)	Protein (aa)	No. of exons (size in bp)	No. of introns (size in bp)
BrAOP2.1 (Bra034180)	1320	439	3 (368 691 261)	2 (293 167)
BrAOP2.2 (Bra000848)	1323	440	3 (371 691 261)	2 (16 881 358)
BrAOP2.3 (Bra018521)	1287	428	3 (371 655 261)	2 (398 853)

**Fig. 2.**
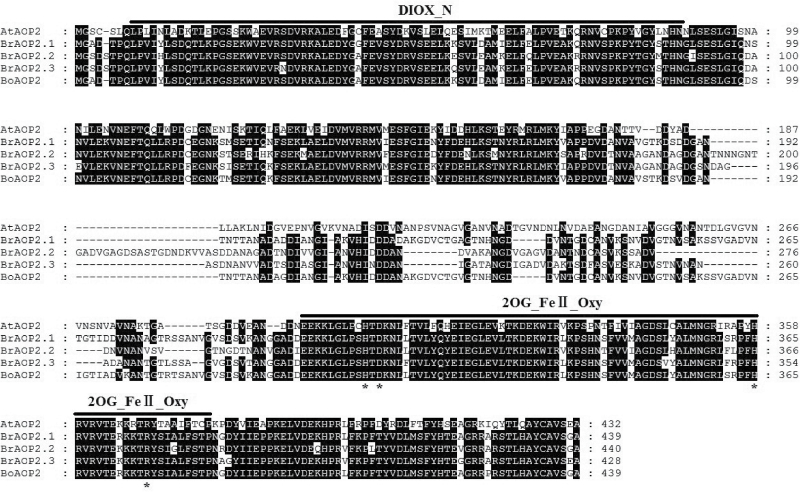
Multiple alignments of the BrAOP2, BoAOP2, and AtAOP2 protein sequences. Multiple alignments were performed using MEGA v.6.0. AtAOP2 is a known functional protein from *Arabidopsis thaliana* (ecotype Cvi), and BoAOP2 is from *B. oleracea* (collard). The solid lines above the alignment indicate the consensus sequences for the DIOX-N and 2OG-FeII_Oxy domains identified using the Conserved Domain Database (Marchler-Bauer *et al.*, 2011)). The black shading indicates that at least four proteins share the same amino acid site. Asterisks show four active-site residues in the 2OG-FeII_Oxy domain.

Alignment of the amino acids confirmed the presence of two conserved domains, DIOX-N and 2OG-FeII_Oxy, at the N-terminal and C-terminal regions of the BrAOP2s, respectively (Fig. 2). These two conserved domains are known to be responsible for 2-oxoglutarate/Fe(II)-dependent dioxygenase activity, which is associated with an important class of enzymes that mediate a variety of oxidative reactions (Prescott and Lloyd, 2000). In contrast to the two highly conserved domains, the middle part of BrAOP2 proteins only showed patches of similarity (Fig. 2). In this variable region, the deduced BrAOP2 proteins were all composed of three motifs (e.g. motif 12, 13, 14), with one additional motif 14 involved in BrAOP2.2 and BrAOP2.3, respectively (Supplementary Fig. S1 and Table S7, available at *JXB* online). The structural divergence of BrAOP2 proteins might have consequences for their functional diversification.

**Fig. 3.**
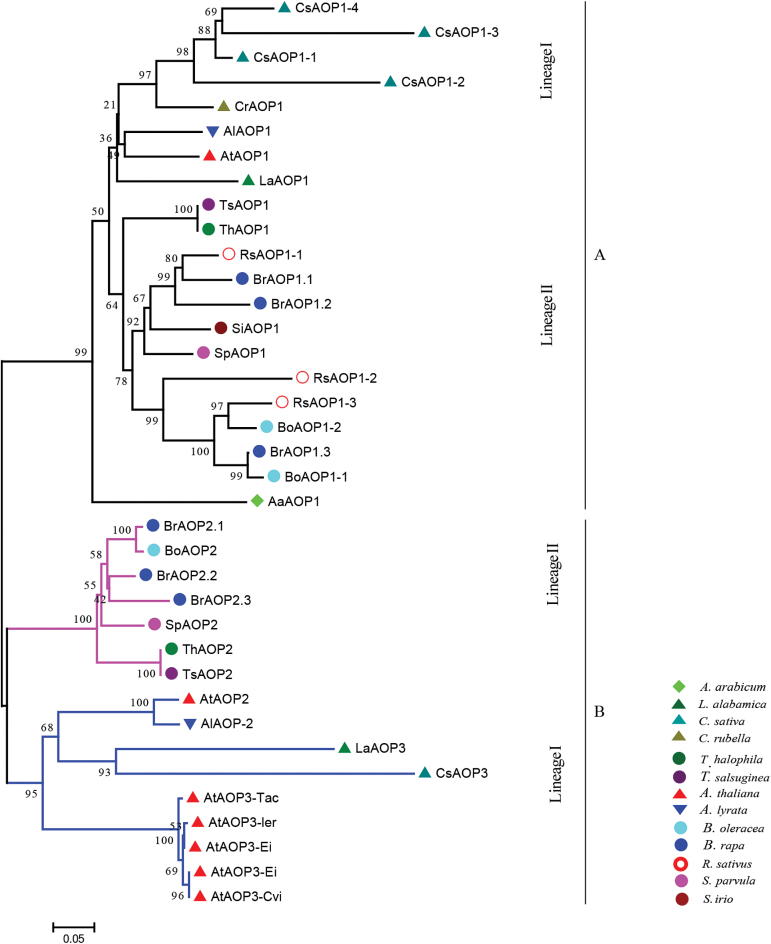
Evolutionary relationships and motif structures of Brassicaceae AOP proteins. Phylogenetic analysis was performed based on the conserved sequences at the N and C terminal parts of AOP proteins using the neighbour-joining method (bootstrap test with 1000 replicates) (Tamura *et al.*, 2011). The sequenced Brassicaceae species in BRAD included: lineage I species *Arabidopsis thaliana*, *Arabidopsis lyrata*, *Capsella rubella*, *Camelina sativa*, and *L. alabamica*; lineage II species *T. halophila*, *T. salsuginea*, *S. parvula* (synonym of *T. parvula*), *S. irio*, *B. rapa*, *B. oleracea*, and *R. sativus*; and an early branching sister of the core Brassicaceae, *Aethionema arabicum*. Black branches indicate AOP1 proteins, coloured branches indicate AOP2 and AOP3 proteins. The tree is drawn to scale; bar, number of substitutions per site.

The Brassicaceae is a medium-sized family that can be split into two major groups: the *Aethionema* group and the core group (Franzke *et al.*, 2011). Three major lineages (I, II, and III) of the core group have been proposed based on the sequences of the chloroplast NADH dehydrogenase gene *ndhF* (Beilstein *et al.*, 2006) and supported by subsequent studies (Koch *et al.*, 2007; Beilstein *et al.*, 2008; Couvreur *et al.*, 2010). To investigate further the evolutionary origin of the three *BrAOP2* genes, we first identified all *AOP* genes in the 13 sequenced Brassicaceae species in BRAD (Cheng *et al.*, 2011) according to their genome annotation information and their syntenic relationship with *Arabidopsis thaliana AOP* genes (http://brassicadb.org/brad/searchSyntenytPCK.php) (Supplementary Table S2, available at *JXB* online). We next reconstructed a phylogenetic tree of all deduced AOP amino acid sequences using the maximum-likelihood method (Tamura *et al.*, 2011) (Fig. 3).

Phylogenetic analyses revealed that all sequenced Brassicaceae species in core Brassicaceae retained *AOP1* genes derived from *AaAOP1* of *Aethionema arabicum* (Fig. 3, group A). Most of the lineage II species (excluding *S. irio* and *R. sativus*) retained *AOP2* genes. Most of the lineage I species possessed *AOP3* genes, which demonstrated further structural divergence in the middle part from *AOP2* (Fig. 3, group B; Supplementary Fig. S1 and Supplementary Table S1). *BrAOP2.1* grouped with *BoAOP2* with high bootstrap support. Although *AtAOP2* and *AtAOP3* clustered together in *Arabidopsis thaliana* accessions, they are not co-expressed because of their inverted gene structure (Chan, *et al.*, 2010). All *AOP2* genes of the genus *Brassica* were more similar to those of the genus *Thellungiella* than the genus *Arabidopsis*.

### The three BrAOP2 proteins are all located in the cytoplasm

**Fig. 4.**
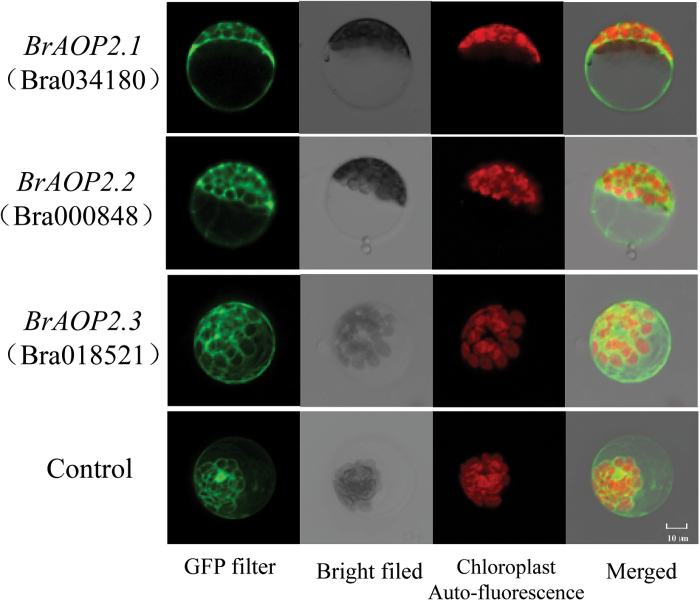
Subcellular localization of BrAOP2 proteins in *B. rapa* protoplasts. Images were taken in a dark field for green fluorescence and chloroplast autofluorescence (red), while the outlook of cells was photographed in a bright field. Bar, 10 µm.

To investigate the subcellular localization of the three BrAOP2 proteins, three Pro_CAMV35S_:BrAOP2:GFP vectors were constructed and their localization was detected by monitoring the transient expression of GFP in *B. rapa* mesophyll protoplast cells (Fig. 4). A strong green fluorescent signal was observed in the cytoplasm of transiently transformed cells, demonstrating that the three BrAOP2 proteins were predominantly cytoplasmic, consistent with the subcellular localization for the secondary modification of aliphatic glucosinolates in *Arabidopsis* (Sonderby *et al.*, 2010).

### The three BrAOP2 paralogues are all active in *B. rapa*

**Fig. 5.**
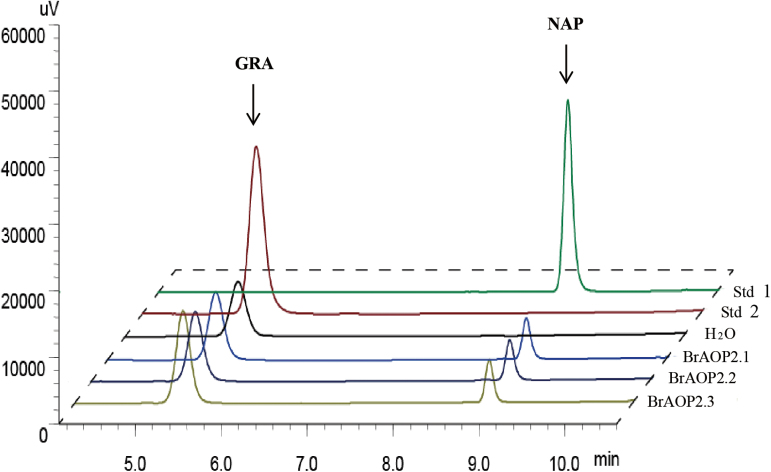
Enzymatic activity of BrAOP2 heterologously expressed in *E. coli*. HPLC results (monitored at 229nm) of purified desulfoglucosinolates from bacterial extracts containing heterologously expressed BrAOP2 fusion proteins is shown. The compound identities were confirmed by comparing retention times and UV light absorption profiles with those of authentic standards. Std 2 indicates desulfated GRA standard, Std 1 indicates desulfated NAP standard, BrAOP2.1, BrAOP2.2, and BrAOP2.3 indicate GRA treated with these three BrAOP2 enzymes, and H_2_O shows GRA treated with ddH_2_O as the negative control.

The *in vitro* catalytic activity of the three BrAOP2 proteins can be monitored readily when they are expressed heterologously by measuring the conversion of GRA to 3-butenyl glucosinolate (NAP). We therefore heterologously expressed and purified thioredoxin fusion proteins for the three *BrAOP2* genes in *E. coli*. As shown in Fig. 5, all three BrAOP2 proteins successfully catalysed the conversion of GRA to NAP, showing that they all had the capacity to convert methylsulfinylalkyl glucosinolates to the alkenyl form (GSL-ALKs).

The *in vivo* functional contribution of each *BrAOP2* gene to the conversion of methylsulfinylalkyl glucosinolate to alkenyl glucosinolates can be assessed in *AOP2* mutant *Arabidopsis* by the complementation test. *Arabidopsis* Col-0 contains a non-functional *AtAOP2* allele with natural variations that lead to the accumulation of GRA (Kliebenstein *et al.*, 2001*b*), which is regarded as an AOP2 mutant in the complementation test. All three *BrAOP2* genes (under the control of the constitutive CaMV 35S promoter) were introduced into *Arabidopsis* Col-0, and at least three independent homozygous lines of each *BrAOP2* gene were analysed for total and individual glucosinolate fractions in 6-week-old rosette leaves.

**Fig. 6.**
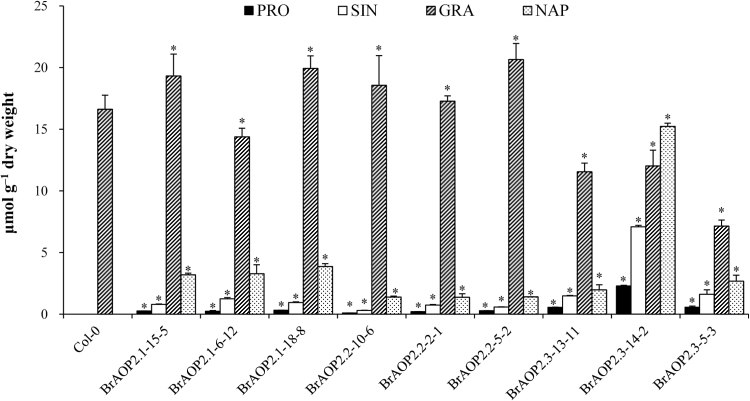
Functional complementation analyses of *BrAOP2* genes in *Arabidopsis thaliana* Col-0. The glucosinolate content and profile were determined in 6-week-old rosette leaves. Three independent mutant-complemented lines for each *BrAOP2* gene were analysed, and average foliar glucosinolates from 30 individual plants are represented along with their standard errors. Asterisks indicate significant differences in glucosinolate content compared with Col-0 (*P*<0.05, one-way ANOVA analysis with a Duncan post-hoc test). PRO, 2-hydroxy-3-butenyl-glucosinolate; SIN, sinigrin.

The functional complementation among the *BrAOP2* genes catalysed the conversion of the C3 and C4 methylsulfinylalkyl glucosinolate to alkenyl glucosinolates (e.g. sinigrin and NAP); this conversion was not observed in the Col-0 control (Fig. 6). Line *BrAOP2.3*-14-2 showed a high level of conversion of the precursor methylsulfinylalkyl glucosinolate to sinigrin and NAP as well as to 2-hydroxy-3-butenyl-glucosinolate (PRO). PRO was further hydroxylated by the action of the GS-OH locus product (Mithen *et al.*, 1995). The two lines *BrAOP2.1*-15-5 and *BrAOP2.1*-18-8 both showed a slightly lower conversion of the precursor methylsulfinylalkyl glucosinolate to sinigrin and NAP. All three *BrAOP2.2* lines showed relatively low conversions of the precursor methylsulfinylalkyl glucosinolate to sinigrin and NAP, producing trace amounts of PRO. Thus, the mutant complementation analysis in *Arabidopsis thaliana* clearly suggested that all three *BrAOP2* genes had biological activities *in vivo*.

### His356, Asp310, and Arg376 residues are required for the catalytic activity of BrAOP2.1

**Fig. 7.**
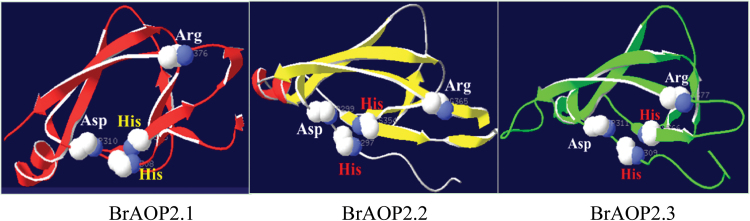
Four active-site residues identified in three BrAOP2 2OG-FeII_Oxy domains. The tertiary structure of each BrAOP2 protein was predicted with the program SWISS-MODEL, and the representation was made with Swiss Pdb-Viewer 4.01 (Arnold et al, 2006). (This figure is available in colour at *JXB* online.)

The two conserved domains DIOX-N and 2OG-FeII_Oxy at the N- and C-terminal regions of BrAOP2 (Fig. 2) are essential for 2-oxoglutarate/Fe(II)-dependent dioxygenase activity (Pfam accession number PF03171), which can act on a variety of substrates (Schofield and Zhang, 1999; Costas *et al.*, 2004). The catalytically active site is formed by a mononuclear non-haem Fe centre co-ordinated by two histidine residues and one carboxylate moiety (Hegg and Que, 1997; Zhou *et al.*, 1998; Neidig *et al.*, 2004; Purpero and Moran, 2007). We identified four active-site residues, His308, His356, Asp310, and Arg376, in the conserved 2OG-FeII_Oxy domain of BrAOP2.1 using the PROSITE database (http://prosite.expasy.org/) (Fig. 7).

**Fig. 8.**
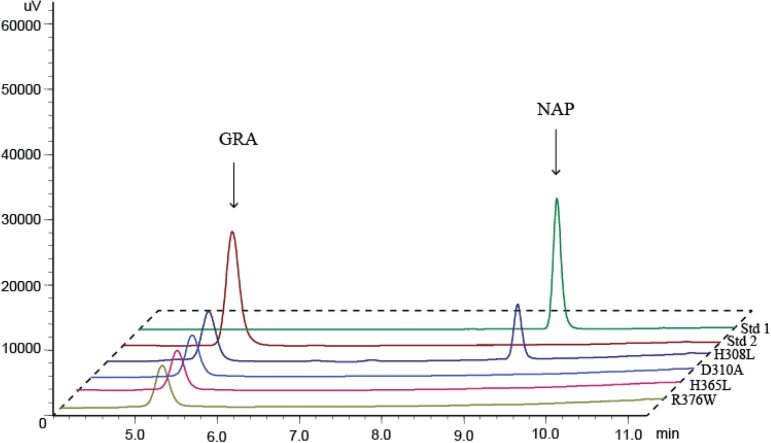
Enzymatic activity of heterologously expressed site-directed mutant BrAOP2.1 proteins. The purified mutant proteins were assayed with GRA and the products were extracted and analysed by HPLC as described in Materials and methods. Site-directed mutagenesis of BrAOP2.1 was conducted four times to test the four active-site residues. (This figure is available in colour at *JXB* online.)

To test the hypothesis that strictly conserved amino acid residues are required for AOP2 activity, we mutated the nucleotides that encode the conserved amino acids of BrAOP2.1 by site-directed mutagenesis. The Asp310 codon was changed to encode alanine (D310A), the histidine codons at positions 308 and 356 were changed to encode leucine (H308L and H356L), and the codons for arginine residue 376 were changed to encode tryptophan (R376W). Heterologous expression and enzyme assays were conducted for the mutant proteins, and the purified H308L mutant enzyme was shown to still catalyse the conversion of GRA to NAP, suggesting that His308 may be not essential for the catalytic mechanism (Fig. 8). By contrast, when His356, Asp310, or Arg376 were changed to leucine, alanine, and tryptophan, respectively, no catalytic activity was detected in any of the mutant BrAOP2.1 proteins (Fig. 8). These results suggested that these three highly conserved amino acid residues shown to be necessary for iron binding in the 2OG-FeII_Oxy domain are crucial for enzymatic activity in BrAOP2.

### *BrAOP2* genes exhibit distinct expression levels in *B. rapa*

Genome polyploidy events are often associated with variable expression of homologous gene pairs within a genome. Expression divergence between duplicate genes has long been of interest to geneticists and evolutionary biologists (Ohno, 1970; Ferris and Whitt, 1979) because divergence is considered the first step in functional divergence between duplicate genes, which increases the chance of retention of duplicate genes in a genome (Ohno, 1970). Indeed, the divergence of gene expression between duplicate genes has been reported in many studies (Blanc and Wolfe, 2004; Casneuf *et al.*, 2006; Duarte *et al.*, 2006; Tuskan *et al.*, 2006).

**Fig. 9.**
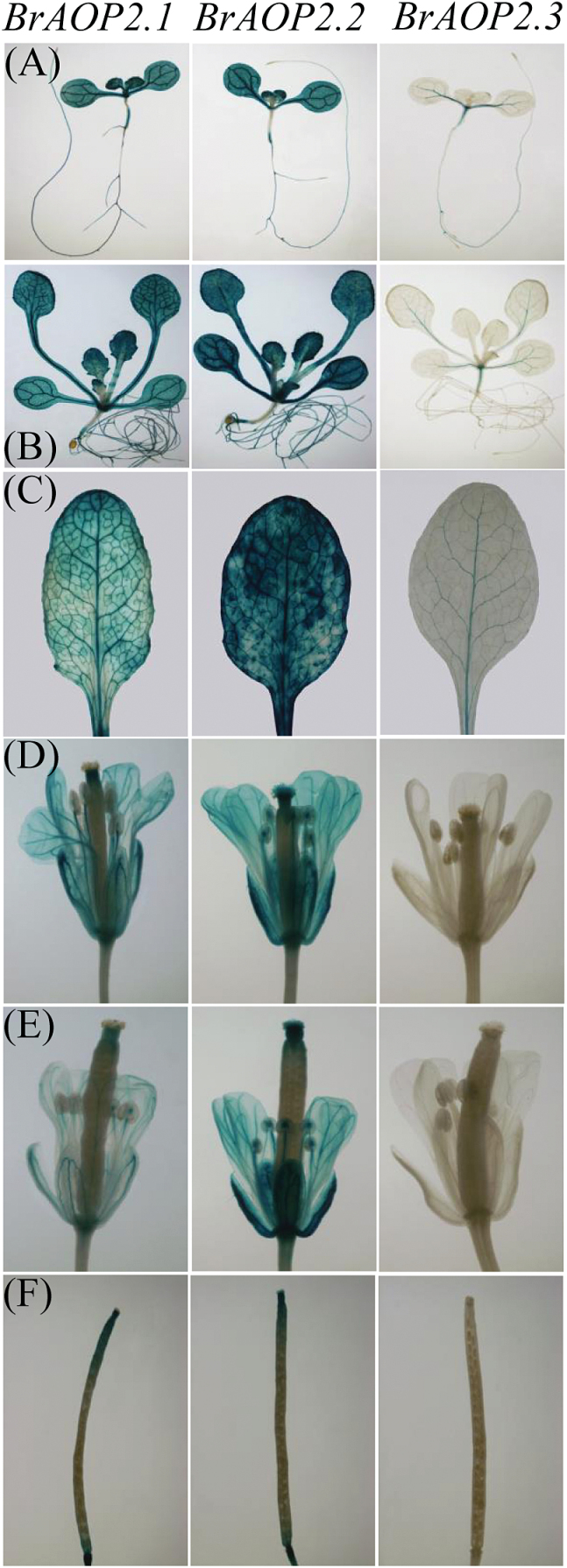
Histochemical GUS staining of Pro_*BrAOP2*_:GUS transgenic *Arabidopsis* lines at different developmental stages. (A) One-week-old seedlings, (B) 3-week-old seedlings, (C) 5-week-old rosette leaves, (D) flowers, (E) immature silique, and (F) mature silique. Plants from stages (A) and (B) were grown on MS medium, and plants from stage (C–F) were grown on soil. A GUS histochemical assay was carried out on three independent single-copy transgenic lines of each *BrAOP2* paralogue in the T3 generation and showed the same result.

To better understand the expression of *BrAOP2* genes, we generated transgenic plants carrying a GUS reporter driven by each *BrAOP2* promoter (Pro_*BrAOP2*_:GUS). Reporter gene expression in transgenic *Arabidopsis* lines revealed that *BrAOP2.1* and *BrAOP2.2* paralogues showed similar and stable expression patterns, while Pro_*BrAOP2.3*_:GUS lines showed distinct expression patterns during the developing stages of *Arabidopsis thaliana* (Fig. 9).

Histochemical analysis revealed higher GUS activity in the seedlings, mature rosette leaves, flowers, and siliques of Pro_*BrAOP2.1*_:GUS transgenic lines compared with Pro_*BrAOP2.2*_:GUS lines. In Pro_*BrAOP2.3*_ transgenic lines, prominent GUS staining was detected in the cotyledon of seedlings and mature rosette leaves (Fig. 9A–C), with undetectable GUS expression in the roots of 1-week-old seedlings, flowers, and siliques. In the rosette leaves of *Arabidopsis*, the *BrAOP2* promoter showed cell-specific GUS activity. This promoter showed maximal activity in the whole leaves, including the vasculature and mesophyll cells, whereas the predominant activity of the *BrAOP2.1* and *BrAOP2.2* promoters was observed in the mid-vein, primary, and secondary veins (Fig. 9B), and that of the *BrAOP2.3* promoter was mainly in the mid-vein and primary veins. The non-uniform and cell-specific expression patterns of the *BrAOP2* genes within leaves, roots, and reproductive tissues might have important implications for the accumulation of NAP across different regions of the plant. Thus, the GUS histochemical data obtained using Pro_*BrAOP2*_:GUS *Arabidopsis* lines confirmed that the *BrAOP2.3* promoter has overlapping but distinct cell and tissue expression patterns.

**Fig. 10.**
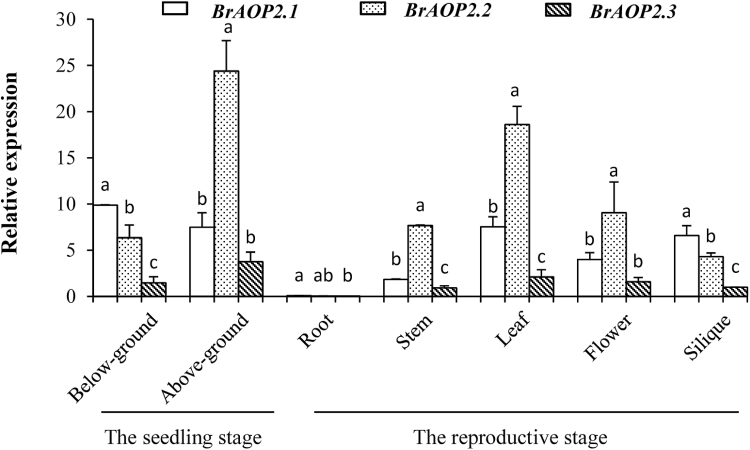
Relative expression profiles of *BrAOP2* genes in L143 organs at the seedling stage (2 weeks) and reproductive stage (10 weeks) (horizontal axis). The vertical axis indicates gene expression levels relative to *BrAOP2.3* (Bra000848) expression in siliques. Error bars represent the standard deviation from three biological repeats. Different lowercase letters indicate significant differences at *P*<0.05 using ANOVA analysis followed by a Duncan post-hoc test.

We further measured *BrAOP2* expression at the transcript level during different developmental stages and in different tissue types in accession L143 (a yellow sarson accession) using qRT-PCR analysis. In general, *BrAOP2.1* showed a slightly different pattern of expression in below-ground tissue at seedling stage and silique at reproductive stage compared to *BrAOP2.2* and *BrAOP2.3* genes (Fig. 10). In the seedling stage, the expression of *BrAOP2.1* was higher than that of *BrAOP2.2* and *BrAOP2.3* in below-ground tissue, while in above-ground tissues the expression of *BrAOP2.2* was higher than that of *BrAOP2.1* and *BrAOP2.3*. In the reproductive stage, the three *BrAOP2* genes were expressed abundantly in glucosinolate-synthesizing tissues such as leaves, siliques, and flowers, as well as in transporting tissue stems; only a trace accumulation of these transcripts was detected in the roots. In above-ground tissues, *BrAOP2.2* was highly expressed in the leaves, stems, and inflorescence, but was less expressed in the siliques. *BrAOP2.1* was abundant in the siliques and leaves, while the expression of *BrAOP2.3* was reduced in all above-ground tissues tested.

## Discussion

Nutritionally and economically important *Brassica* crops such as cabbage, broccoli, and cauliflower are rich sources of glucosinolates, which are present in all organs, especially aliphatic glucosinolates. Here, we have reported the characterization, functional analysis, and expression of *BrAOP2* paralogues in *B. rapa*. The three *BrAOP2* genes exhibited overlapping but distinct tissue- and cell-specific expression patterns, suggesting that they play a co-ordinated role in catalysing the conversion of beneficial GRA to the harmful gluconapin. This information will be helpful to breeders aiming to modify glucosinolate levels in *B. rapa*.

### All three *BrAOP2* genes catalysed the conversion of GRA to NAP

Genome polyploidization is widespread in the plant kingdom where it plays a major role in generating diversity and providing abundant genetic material for the evolution or expansion of gene families and the formation of new genes (Hittinger and Carroll, 2007; Spillane *et al.*, 2007). The evolutionary consequences of duplicated genes after polyploidy include loss or silencing (non-functionalization), retention of the original function, or functional divergence through subfunctionalization or neofunctionalization (Lynch, 2000; Zhang, 2003).

In our study, overexpression of the *BrAOP2* paralogues in *Arabidopsis thaliana* Col-0 and *E. coli* demonstrated that the three encoded BrAOP2 proteins are involved in the conversion of methylsulfinylbutyl glucosinolates to corresponding alkenyl glucosinolates. The enzyme activity suggested that the three *BrAOP2* genes might be involved in a similar reaction in *B. rapa*. Multiple alignments of the amino acid sequences of the three BrAOP2 proteins showed significant structural conservation in the C- and N-terminal regions. However, a frame-shift mutation in exon 2 results in an absent or incomplete 2OG-FeII_Oxy domain (at the N terminus), giving rise to a non-functional *AOP2* allele in other Brassicaceae (e.g. *BoAOP2* from broccoli and *AtAOP2* from Col-0; Li and Quiros, 2003; Neal *et al.*, 2010). Site-directed mutagenesis analyses showed that all three BrAOP2 proteins shared three key active-site residues in their 2OG-FeII_Oxy domains that are crucial for enzymatic activity. Therefore, the 2OG-FeII_Oxy domain and its key active-site residues may be responsible for BrAOP2s retaining their dioxygenase activity across plant development stages and tissue/cell types, and under variable environmental conditions. Thus, polyploidization of the *Brassica* genomes does not seem to have altered the basic *BrAOP2* gene function, and all *AOP2* homologues seem to have retained the subdivision of gene function in most polyploidy *Brassica* crops.

### AOP variation and speciation in the Brassicaceae

The Brassicaceae contains 338 genera and 3709 species, including many economically important crops (Warwick *et al.*, 2006). The core group has undergone three ancient whole-genome duplication events (Franzke *et al.*, 2011), that have played a crucial role in the genetic diversification and species radiation of lineages in the Brassicaceae. Furthermore, whole-genome triplication events have occurred in *Brassica* (Br-α), *L. alabamica* (La-α), and *Camelina sativa* (Cs-α), as determined by analyses of their recently sequenced genomes (Haudry *et al.*, 2013; Slotte *et al.*, 2013; Cheng *et al.*, 2014). Additionally, the genomes of 13 crucifer species have been completely or partially sequenced, and this has provided the opportunity to clarify the evolution of *AOP* genes.

Our phylogenetic analyses showed that the core Brassicaceae species have retained *AOP1*, while *AOP2* is retained by most of the lineage II species (excluding *S. irio* and *R. sativus*), and *AOP3* by lineage I species. The variation in *AOP2*/*AOP3* has led to different aliphatic glucosinolate profiles in each lineage (Al-Shehbaz and Al-Shammary, 1987). Our phylogenetic tree is consistent with the finding that two different gene duplication events occurred in the Brassicaceae *AOP* locus (Kliebenstein *et al.*, 2001*b*); the first event led to the separation of *AOP1* and the *AOP2/AOP3* progenitor, while the second gave rise to the formation of *AOP2* and *AOP3*. Following gene duplications, the separate copies evolved new enzymatic functions. Thus, it is likely that some copies were lost when the genetic backgrounds of species altered, or as a result of environmental adaptation. The variations in lineage specificity of *AOP2* versus *AOP3* in Brassicaceae species agrees with previous data showing that *GS-ALK* (*AOP2*)/*GS-OHP* (*AOP3*) variation occurred in the distantly related *Arabidopsis* and in the genera *Thlaspi* and *Malcolmia* (Daxenbichler *et al.*, 1991). In the current study, the lineage-specific evolution of *AOP* in the core Brassicaceae group was assumed to proceed alongside the split of the three major lineages, and may have been driven by the whole-genome duplication and maintained by environmental pressure.

### Expression divergence of *BrAOP2* genes in *B. rapa*

Studies have shown that gene duplication enables duplicates to become specialized in various organs of the plant or in response to different environmental stimuli (Ferris and Whitt, 1979; Hughes, 1994; Adams, 2007; Ha *et al.*, 2007; Kliebenstein, 2008). Thus, duplicate copy genes would be expected to have more diversified expression profiles than single-copy genes in a group of closely related organisms (Li *et al.*, 2005). Polyploidy has considerable effects on duplicate gene expression, including silencing and up- or downregulation of one of the duplicated genes through increased variation in dosage-regulated gene expression, altered regulatory interactions, and rapid genetic and epigenetic changes (Osborn *et al.*, 2003; Adams and Wendel, 2005).

In the current study, the expression profiles of three *BrAOP2* paralogues were shown to vary in different organs of *B. rapa* (Fig. 10), indicating that they are differentially regulated during plant development. For example, in the L143 reproductive stage, only trace expression of *BrAOP2* was detected in the roots, but expression was abundant in other organs, similar to the expression profiles reported previously for *AtAOP2* (Neal *et al.*, 2010). The histochemical analysis of GUS activity revealed that the three *BrAOP2* genes showed non-uniform and cell-specific expression patterns during the development stages of *Arabidopsis* (Fig. 9). When an approximately 1.2kb region of the sequence upstream of the three *BrAOP2* genes was scanned using the Plant Cis-Acting Regulatory Elements database (Lescot *et al.*, 2002), several specific *cis*-regulatory elements related to tissue-dependent expression and elements responsive to auxin, glucose signalling, and abiotic and biotic responses were observed (see Supplementary Table S8, available at *JXB* online). The disparity of *cis*-regulatory elements observed among the three *BrAOP2* promoters could contribute to the differential expression patterns of *BrAOP2* (Chen *et al.*, 2010). Moreover, the distinct developmental and spatial expression patterns of *BrAOP2* within *Arabidopsis* also suggest a role in distributing glucosinolate content in *B. rapa*, which in turn may have important consequences for plant defence against environmental stresses.

### *BrAOP2*: a crucial candidate for engineering beneficial glucosinolates in *B. rapa*

The hydrolytic breakdown products of GRA, especially the isothiocyanates, are beneficial bioactive constituents that have cancer-preventative properties in humans. However, GRA can further react with the goitrogenic compound progoitrin, which has other detrimental effects on animal health. Therefore, the enrichment of beneficial glucosinolates and the reduction of detrimental glucosinolates has been the focus of much attention in the breeding programmes of *Brassica* crops.

In the current study, we found that NAP was predominant in the total glucosinolate contents of *B. rapa*, which is consistent with the results of other studies (Padilla *et al.*, 2007; Lou *et al.*, 2008; Kim *et al.*, 2010). Three functional *BrAOP2* genes, each encoding an AOP2 enzyme, were identified as giving rise to the abundant NAP found in *B. rapa*. In *B. oleracea*, GRA accumulation was found to be caused by non-functional *GSL-ALK* genes (Li and Quiros, 2003), which are homologous to the *AOP* genes in *Arabidopsis thaliana* (Neal *et al.*, 2010). In *B. oleracea*, a 2bp *GSL-ALK* deletion caused the gene to become non-functional, which abolished the conversion of methylsulfinyl glucosinolates into alkenyl glucosinolates, resulting in an enrichment of GRA (Li and Quiros, 2003). In *B. napus*, the application of a new germplasm with reduced detrimental glucosinolates and increased beneficial glucosinolates was achieved by using RNA interference to downregulate the expression of *GSL-ALK* (Liu *et al.*, 2012). Thus, it is possible to block the side-chain modification to produce GRA-enriched *B. rapa* vegetables.

The work described in this study has helped increase our understanding of *BrAOP2* genes in glucosinolate side-chain modifications of polyploidy *B. rapa*. Our findings provide evidence of expression partitioning and the evolution of *AOP2* gene family homologues in determining the aliphatic glucosinolate profile of *B. rapa*. The information reported here should facilitate the improvement of aliphatic glucosinolate traits in *Brassica* crops.

## Supplementary data

Supplementary data are available at *JXB* online.

Supplementary Fig. S1. Motif structures of *AOP* genes in Brassicaceae.

Supplementary Table S1. Amino acid sequence identity (%) of AOP2 among *Arabidopsis thaliana*, *B. rapa*, and *B. oleracea.*

Supplementary Table S2. *AOP* genes identified in 13 Brassicaceae species in the Brassica Database.

Supplementary Table S3. *BrAOP2* primers used to clone the coding sequences for heterologous expression vector construction.

Supplementary Table S4. Mutagenic primers used to construct mutant *BrAOP2* genes.

Supplementary Table S5. *BrAOP2* primers used to clone the promoter sequences for vector construction.

Supplementary Table S6. Primer sequences for *BrAOP2* and *BrGAPDH* used in qRT-PCR analysis.

Supplementary Table S7. The sequence of motifs in the middle part of AOP2 and AOP3 identiﬁed by MEME.

Supplementary Table S8. Summary of *cis*-regulatory elements present within a 1.2kb upstream region of *BrAOP2* homologues.

Supplementary Data

## Abbreviations:

ANOVA: analysis of variance
CaMV: cauliflower mosaic virus
GAPDH: glyceraldehyde 3-phosphate dehydrogenase
GFP: green fluorescent protein
GRA: glucoraphanin
GSL-ALK: alkenyl form of methylsulfinylalkyl glucosinolate
GUS: β-glucuronidase
HPLC: high-performance liquid chromatography
MS: Murashige and Skoog
NAP: 3-butenyl glucosinolate
PRO: 2-hydroxy-3-butenyl-glucosinolate
qRT-PCR: quantitative real-time reverse transcription-PCR.
