# Supplementary material for: Three genes encoding AOP2, a protein involved in aliphatic glucosinolate biosynthesis, are differentially expressed in Brassica rapa
Source: J Exp Bot. 2015 Jul 17;66(20):6205–18. doi: 10.1093/jxb/erv331 (PMC4588880; doi:10.1093/jxb/erv331)
Supplement: Supplementary Data [file supp_66_20_6205__index.html]

Three genes encoding AOP2, a protein involved in aliphatic glucosinolate biosynthesis, are differentially expressed in Brassica rapa — Three genes encoding AOP2, a protein involved in aliphatic glucosinolate biosynthesis, are differentially expressed in Brassica rapa — Supplementary Data 

# Three genes encoding AOP2, a protein involved in aliphatic glucosinolate biosynthesis, are differentially expressed in *Brassica rapa*

## Supplementary Data

Data files

- Supplementary Data - Supplementary Data
