# Supplementary material for: Three genes encoding AOP2, a protein involved in aliphatic glucosinolate biosynthesis, are differentially expressed in Brassica rapa
Source: J Exp Bot. 2015 Jul 17;66(20):6205–18. doi: 10.1093/jxb/erv331 (PMC4588880; doi:10.1093/jxb/erv331)
Supplement: Supplementary Data [file supp_erv331_jexbot153494_file001.pdf]

Supplementary Data

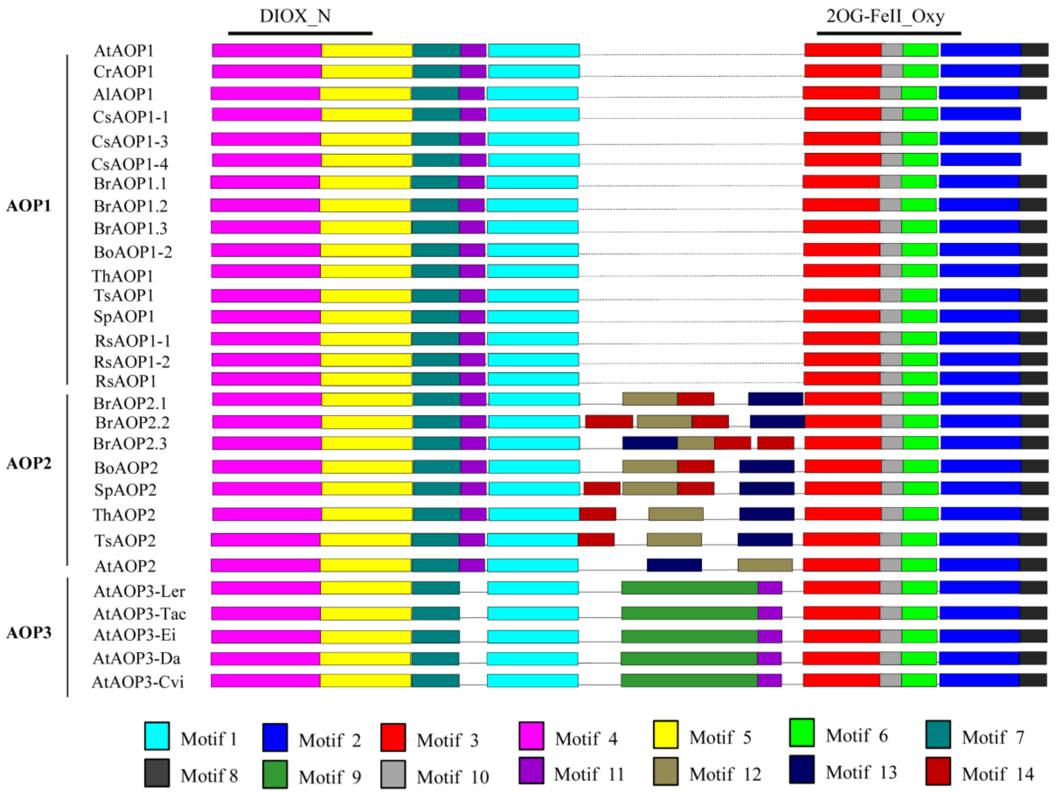

Fig. S1 Motif structures of AOP genes in Brassicaceae. Different colored boxes show protein motifs. At the top, DIOX_N and 2OG-FeII_Oxy indicate two conserved domains at the N-terminal and C-terminal regions of AOP proteins.

Table S1 Amino acid sequence identity (%) of *AOP2* among *A. thaliana*, *B. rapa* and *B. oleracea*

	AtAOP2	*BrAOP2.1*	*BrAOP2.2*	*BrAOP2.3*	*BoAOP2*
AtAOP2	-	60	56	55	59
BrAOP2.1		-	74	78	96
BrAOP2.2			-	81	74
BrAOP2.3				-	80
BoAOP2					-

Table S2 *AOP* genes identified in 13 Brassicaceae species in the Brassica Database

**Species**	**Gene ID**	**Reference**
A. arabicum	*AaAOP1*(AA_scaffold905_70)	(Haudry et al., 2013)
C. rubella	*CrAOP1*(Carubv10003282m)	(Slotte et al., 2013)
L. alabamica	*LaAOP1*(LA_scaffold1283_7)<br>*LaAOP3*(LA_scaffold1283_8)	(Haudry et al., 2013)
C. sativa	*CsAOP1-1*(Csa02g007420.1)<br>*CsAOP1-2*(Csa13g054180.1)<br>*CsAOP1-3*(Csa13g054190.1)<br>*CsAOP1-4*(Csa08g050250.1)<br>*CsAOP3*(Csa02g006400.1)	(Kagale et al., 2014)
A. thaliana	*AtAOP1*(AT4G03070)<br>*AtAOP2* (At4G03060)<br>*AtAOP3*-Ler (AF417859)<br>*AtAOP3*-Tac (AF418282)<br>*AtAOP3*-Ei (AF418276)<br>*AtAOP3*-Da (AF418275)<br>*AtAOP3*-Cvi (AF418274)	(Kliebenstein et al.,2001)
A. lyrata	*AlAOP1* (AF417857.1)<br>*AlAOP-2*(fgenesh2_kg.6_3391_AT4G03060.1)	(Kliebenstein et al., 2001)
T. halophila	*ThAOP1*(Thhalv10028799m)<br>*ThAOP2*(Thhalv10028690m)	(Yang et al., 2013)

T. salsuginea	*TsAOP1*(Tsa6g03220)<br>*TsAOP2*(Tsa6g03210)	(Wu et al., 2012)
S. parvula	*SpAOP1*(c0011_00285)<br>*SpAOP2*(c0011_00284)	(Dassanayake et al., 2011)
S. irio	*SiAOP1*(SI_C230221_4)	(Haudry et al., 2013)
B. rapa	*BrAOP1.1*(Bra034182)<br>*BrAOP1.2*(Bra034181)<br>*BrAOP1.3*(Bra000847)<br>*BrAOP2.1*(Bra034180)<br>*BrAOP2.2*(Bra000848)<br>*BrAOP2.3*(Bra018521)	(Wang et al., 2011)
B. oleracea	*BoAOP1-1*(Bol030626)<br>*BoAOP1-2*(Bol030627)	(Liu et al., 2014)
R. sativus	*RsAOP1-1*(Rsa10009537)<br>*RsAOP1-2*(Rsa10022481)<br>*RsAOP1-3*(Rsa10022483)	(Kitashiba et al., 2014)

Table S3 Primer sequences of *BrAOP2*genes used in coding sequences cloning and heterologous expression vector construction

**Gene ID**	**Primer sequence(5'-3')**
BrAOP2.1(Bra034180) F + NcoI	CATGCC ATGGGTGCAGACACTCC
BrAOP2.1(Bra034180) R+ XhoI	CTCGAG TTATGCTCCAGAGACG
BrAOP2.2(Bra000848) F+ NcoI	CATGCC ATGGGTTCAGACAGTAC
BrAOP2.2(Bra000848) R+ XhoI	CTCGAG TTAAGCTCCGGAGACG
BrAOP2.3(Bra018521) F+ NcoI	CATGCC ATGGGTTCAGACAGTAC
BrAOP2.3(Bra018521) R+ XhoI	CTCGAG TCATGCTTCAGAGACGG

The nucleotide sequences of restriction endonuclease sites are underlined.

Table S4 Mutagenic primers used to construct the mutant *BrAOP2* genes

**Mutant enzyme**	**Oligonucleotide(5’-3’)**	**Codon change**
H308L	GTCTACCTTCT**CTT**ACTGATAAAAACCTTTTG<br>CAGATGGAAGA**GAA**TGACTATTTTTGGAAAAC	CAT-CTT
D310A	GTCTACCTTCTCTTACT**GCT**AAAAACCTTTTG<br>CAGATGGAAGAGAATGA**CGA**TTTTTGGAAAAC	GAT-GCT
H308L	GTCTCGTCCGTTT**CTT**AGAGTAAGAGTAAC<br>CAGAGCAGGCAAA**GAA**TCTCATTCTCATTG	CAT-CTT
R376W	GAAAGAAGACA**TGG**TATTCAATAGC<br>CTTTCTTCTGT**ACC**ATAAGTTATCG	AGG-TGG

The table shows the nucleotide sequences of the coding strand of the wild-type gene that surrounds the numbered residue and the complementary oligonucleotides used to create the indicated mutant genes. The histidine, aspartic acid, and arginine codons are printed in bold and the altered bases are underlined.

Table S5 *BrAOP2* primers used to clone the promoter sequences for vector construction

**Gene ID**	**Primer sequence(5’-3’)**
BrAOP2.1 Pro F + PstI	AAAACTGCAG GGAAGCAGCCGTAG
BrAOP2.1 Pro R+ XbaI	GCTCTAGA TCTTTTCGCTGCAAATC
BrAOP2.2 Pro F+ PstI	AAAACTGCAG GAATTAATATTTTGGCCC
BrAOP2.2 Pro R+ XbaI	GCTCTAGA TCTTCTTTCCTTTGGTG
BrAOP2.3 Pro F+ HindIII	GCCAAGCTT CTACGCATGCGTAACGCTC
BrAOP2.3 Pro R+ XbaI	GCTCTAGA TAGATCACTGGAAGTTGGG

The nucleotide sequences of restriction endonuclease sites are underlined.

Table S6 Primer sequences of the *BrAOP2* and *BrGAPDH* genes used in the qRT-PCR analysis

**Gene ID**	**Primer sequence(5’-3’)**
BrAOP2.1 F	AAGAGTATGAGCGAAACGATCCAG
BrAOP2.1 R	CAGCATCAGCGTTAGCAGTTG
BrAOP2.2 F	AACCTTGTCATTGTCACCAGT
BrAOP2.2 R	CAAGAGTACCAGCGAAAGGAT
BrAOP2.3 F	ACAAGAGTATCAGCGAAACAATCC
BrAOP2.3 R	ACTAGTATCAGCAACAACATTGGC
BrGAPDH(Bra016729) F	CCACTTGCCAAGGTTATCAACGAC
BrGAPDH(Bra016729) R	CAACTGAAACATCAACGGTGGG

Table S7 The sequence of motifs in the middle part of AOP2 and AOP3 identified by MEME

Motif	Width	Best possible match
9	80	ITAAVGANVDNGANDNADGDANVNDDGASIGVKVNVDVGDDVNDNDS VNIGVGVDINVETNVNGDLDAEANGDATAWVVG
11	8	HGNKSISE
12	29	NADVNDIANGIANVHIDDDANAGANACVG
13	29	NADVNANAGTRTSANVGVGDSVKANGGAD
14	21	NHNGDDVNTSDCASVKSNVDV

Table S8 Summary of various cis-regulatory elements present within 1.2 kb upstream region of *BrAOP2* homologs, obtained using PLACE database (www.dna.affrc.go.jp/PLACE/).

cis-elements	BrAOP2.1	BrAOP2.2	BrAOP2.3
-10PEHVPSBD	1	1	2
-300ELEMENT		2	2
-300CORE			1
2SSEEDPROTBANAPA	1		
ABRELATERD1	1	3	4
ACGTATERD1	2	6	8
ABRERATCAL		3	2
ACGTTBOX		2	2
AGCBOXNPGLB		1	
AMYBOX1	1	1	1
ANAERO1CONSENSUS	5	2	1
ARR1AT	18	12	9
ARFAT			1
AUXREPSIAA4	1		
ASF1MOTIFCAMV			1
BIHD1OS	2	3	
BOXIINTPATPB	2	1	1
CAATBOX1	8	14	13
CACGTGMOTIF		2	2
CACTFTPPCA1	17	15	12
CANBNNAPA	1	1	
CAREOSREP1		1	1
CARGCW8GAT	2	4	
CATATGGMSAUR	4	4	
CBFHV			2
CCA1ATLHCB1		2	1
CCAATBOX1			2
CGCGBOXAT		2	
CEREGLUBOX2PSLEGA			1
CGACGOSAMY3			2
CIACADIANLELHC	1		3
CRTDREHVCBF2			2
CURECORECR			6
DOFCOREZM	34	21	30
DPBFCOREDCDC3	2	2	2
DRE1COREZMRAB17	1		1
E2FCONSENSUS			1
EBOXBNNAPA	10	14	8

EECCRCAH1	5	3	3
ELRECOREPCRP1	1		
ERELEE4	3	2	2
GAREAT	1	1	1
GATABOX	12	9	8
GCCCORE	1	1	
GT1CONSENSUS	22	9	16
GT1CORE	1		2
GT1GMSCAM4	7	3	6
GTGANTG10	5	11	9
HEXAMERATH4			1
HEXMOTIFTAH3H4			1
IBOXCORE	3		5
INRNTPSADB	1	3	1
LTRE1HVBLT49	2		
IRO2OS		1	1
L1BOXATPDF1		2	1
LECPLEACS2		1	
MARABOX1	1		
MARTBOX	5	1	3
MYB1AT	3	3	6
MYB2AT	2		
MYB1LEPR		1	1
MYB2CONSENSUSAT	2	1	1
MYBCORE	2	2	2
MYBCOREATCYCB1		1	1
MYBGAHV	1	1	1
MYBPLANT			1
MYCATERD1	2		1
MYCATRD22	2		1
MYBST1		2	
MYCCONSENSUSAT	10	14	8
NODCON1GM	3	1	6
NODCON2GM	2	3	
NTBBF1ARROLB	2	1	
OSE1ROOTNODULE	5	4	6
P1BS		4	
PALBOXAPC		1	
POLASIG1	2	7	4
POLASIG2	2	5	1
POLASIG3	3	8	2
POLLEN1LELAT52	16	6	11
PREATPRODH	1		1

PYRIMIDINEBOXHVEPB1	1		
PYRIMIDINEBOXOSRAMY1A	3		3
QELEMENTZMZM13	1		
RAV1AAT	4	3	7
RBCSCONSENSUS	1		1
REALPHALGLHCB21	1	1	4
RHERPATEXPA7		1	2
ROOTMOTIFTAPOX1	9	11	5
SEF1MOTIF	1		
RYREPEATBNNAPA			1
SEF3MOTIFGM	1		
S1FBOXSORPS1L21			1
SBOXATRBCS			1
SEBFCONSSTPR10A		2	1
SEF3MOTIFGM		1	2
SEF4MOTIFGM7S	3	4	5
SITEIIATCYTC			1
SORLIP1AT	1		1
SORLIP2AT			1
SORLREP3AT		1	
SURECOREATSULTR11	1	2	4
SV40COREENHAN			1
TAAAGSTKST1	6	5	5
TATABOX2	3	4	1
TATABOX3		2	
TATABOX4	1	5	
TATABOX5	4	4	2
TBOXATGAPB	3		3
TGTCACACMCUCUMISIN	1		
TATABOXOSPAL		1	
TATAPVTRNALEU		2	
TATCCAOSAMY		1	
TGTCACACMCUCUMISIN		1	
TGACGTVMAMY			1
WBBOXPCWRKY1	1		
WBOXATNPR1	3		2
WBOXHVISO1	1		2
WBOXNTERF3	3	1	2
WRKY71OS	5	4	3
